# The association between vaginal bacterial composition and miscarriage: a nested case–control study

**DOI:** 10.1111/1471-0528.15972

**Published:** 2019-10-31

**Authors:** M Al‐Memar, S Bobdiwala, H Fourie, R Mannino, YS Lee, A Smith, JR Marchesi, D Timmerman, T Bourne, PR Bennett, DA MacIntyre

**Affiliations:** ^1^ Tommy’s National Centre for Miscarriage Research Imperial College London London UK; ^2^ Imperial College Parturition Research Group Division of the Institute of Reproductive and Developmental Biology Imperial College London London UK; ^3^ Queen Charlotte’s Hospital Imperial College Healthcare NHS Trust London UK; ^4^ March of Dimes European Preterm Birth Research Centre Imperial College London London UK; ^5^ School of Medicine Cardiff University Cardiff UK; ^6^ Division of Integrative Systems Medicine and Digestive Disease Imperial College London London UK; ^7^ School of Biosciences Cardiff University Cardiff UK; ^8^ Department of Obstetrics and Gynaecology University Hospitals Leuven KU Leuven Leuven Belgium

**Keywords:** First trimester miscarriage, second trimester miscarriage, vaginal bacteria, vaginal microbiome

## Abstract

**Objective:**

To characterise vaginal bacterial composition in early pregnancy and investigate its relationship with first and second trimester miscarriages.

**Design:**

Nested case–control study.

**Setting:**

Queen Charlotte’s and Chelsea Hospital, Imperial College Healthcare NHS Trust, London.

**Population:**

161 pregnancies: 64 resulting in first trimester miscarriage, 14 in second trimester miscarriage and 83 term pregnancies.

**Methods:**

Prospective profiling and comparison of vaginal bacteria composition using 16S rRNA gene‐based metataxonomics from 5 weeks’ gestation in pregnancies ending in miscarriage or uncomplicated term deliveries matched for age, gestation and body mass index.

**Main outcome measures:**

Relative vaginal bacteria abundance, diversity and richness. Pregnancy outcomes defined as first or second trimester miscarriage, or uncomplicated term delivery.

**Results:**

First trimester miscarriage associated with reduced prevalence of *Lactobacillus* spp.‐dominated vaginal microbiota classified using hierarchical clustering analysis (65.6 versus 87.7%; *P *= 0.005), higher alpha diversity (mean Inverse Simpson Index 2.5 [95% confidence interval 1.8–3.0] versus 1.5 [1.3–1.7], *P *= 0.003) and higher richness 25.1 (18.5–31.7) versus 16.7 (13.4–20), *P *= 0.017), compared with viable pregnancies. This was independent of vaginal bleeding and observable before first trimester miscarriage diagnosis (*P *= 0.015). Incomplete/complete miscarriage associated with higher proportions of *Lactobacillus* spp.‐depleted communities compared with missed miscarriage. Early pregnancy vaginal bacterial stability was similar between miscarriage and term pregnancies.

**Conclusions:**

These findings associate the bacterial component of vaginal microbiota with first trimester miscarriage and indicate suboptimal community composition is established in early pregnancy. While further studies are required to elucidate the mechanism, vaginal bacterial composition may represent a modifiable risk factor for first trimester miscarriage.

**Tweetable abstract:**

Vaginal bacterial composition in first trimester miscarriage is associated with reduced *Lactobacillus* spp. abundance and is independent of vaginal bleeding.

## Tweetable abstract

Vaginal bacterial composition in first trimester miscarriage is associated with reduced *Lactobacillus* spp. abundance and is independent of vaginal bleeding.

## Introduction

Miscarriage is the most common adverse pregnancy outcome, complicating up to 25% of pregnancies.^1^ Around 50% of all miscarriages are thought to be due to aneuploidy or other chromosomal aberrations, with the causal drivers of the remaining cases largely unknown.^1^ However, evidence supports an infectious aetiology in both first and second trimester miscarriage.^2^ Earlier studies using non‐objective, symptomatic measures suggested infection as a rare cause of pregnancy loss,^3^, ^4^ but a recent study reported histological chorioamnionitis in 78/101 (77%) of miscarriage samples compared with 0/103 (0%) of controls, with 47% of chorioamnionitis cases culture‐positive.^5^ Moreover, a relation between bacterial vaginosis (BV) and increased risk of miscarriage^6^, ^7^, ^8^, ^9^ has been described and chlamydial infection is thought to cause miscarriage through impairment of endometrial decidualisation.^10^, ^11^

Recent application of culture‐independent methods for the assessment of vaginal microbial composition has shown that healthy pregnancy is characterised by stable vaginal bacterial composition with *Lactobacillus* spp. dominance and low bacterial richness and diversity.^12^, ^13^, ^14^ Increased vaginal microbiota stability in pregnancy has been partly attributed to increased estrogen levels, which are thought to stimulate glycogen deposition in vaginal epithelial cells favouring colonisation of *Lactobacillus* spp., as well as decreased sexual activity, hygiene changes and the lack of cyclic hormonal variations.^15^, ^16^ In contrast, adverse pregnancy outcomes such as preterm pre‐labour rupture of membranes and preterm birth are associated with reduced *Lactobacillus* spp. dominance and a shift towards high bacterial diversity community compositions.^17^, ^18^, ^19^, ^20^, ^21^, ^22^ However, vaginal microbial composition is poorly described prior to 8 weeks of gestation, a crucial time period of pregnancy given that most miscarriages occur early in the first trimester.^23^, ^24^

We hypothesised that vaginal bacterial instability and *Lactobacillus* spp. depletion may be associated with miscarriage. We tested this hypothesis by comparing early vaginal bacterial composition in 161 pregnancies of which 64 resulted in first trimester miscarriage, 14 in second trimester miscarriage and 83 healthy term delivery.

## Methods

### Study design and ethical approval

This study was a prospective observational cohort study based at Queen Charlotte’s & Chelsea Hospital, London, between March 2014 and March 2016. The study was approved by NHS National Research Ethics Service (NRES) Riverside Committee London (REC 14/LO/0199), and all participants provided written informed consent. Patients were not involved in the development of the research.

Women in the first trimester of pregnancy with an intrauterine pregnancy were invited to participate. The first trimester was defined as <14 weeks’ gestation by last menstrual period (LMP) or, where LMP was not known, ultrasound scan dating based on crown‐rump length measurements (CRL).^25^ An intrauterine pregnancy was defined on the basis of an ultrasound scan showing an intrauterine gestation sac with or without a visible embryo and heartbeat. Participants were recruited via open advertisements (using posters) in local GP surgeries, in local hospitals and at the university where the study is being conducted (Imperial College). The majority of women were recruited after attending the hospital Ultrasound Department or Early Pregnancy Assessment Unit. Exclusion criteria for this study included women under 16 years of age, multiple pregnancies, sexual intercourse within 72 hours of sampling and human immunodeficiency virus (HIV) or Hepatitis C‐positive status.

A detailed questionnaire including details on demographic information, past medical, gynaecological and obstetric history was completed. Validated symptom scores were used at each study visit to assess vaginal bleeding based upon a pictorial blood assessment chart score at the time of sampling.^26^ Ethnicity was self‐reported as White, Black or Asian. Depending on the gestational age at the time of recruitment and clinical need, participants were seen a minimum of twice and up to five times in the first trimester during pregnancy. Serial ultrasound scans were performed until the end of the first trimester with routine ultrasonographic measurements collected at each visit. Participants were encouraged to contact the research team if they had any complications, such as bleeding, and when necessary were invited to attend for an additional ultrasound scan with the team. Participants were subsequently seen at the time of their routine dating scan (11–14 weeks’ gestation).

### Vaginal sampling

Cervicovaginal fluid samples were collected from each participant from the posterior vaginal fornix using a BBL CultureSwab MaxV Liquid Amies swab (Becton, Dickinson and Company, Oxford, UK) within the gestational time windows of 5–8, 8–10, and 10–14 and >14 weeks’ gestation. Swabs were placed immediately on ice before being frozen and stored at −80°C within 5 minutes of collection. Negative control swabs were also collected by exposing swabs to clinic and laboratory environments prior to freeze storage.

### Pregnancy outcomes

Pregnancy outcomes were collected and documented using hospital medical records Cerner Millennium® and Powerchart®. The core outcome sets were miscarriage and live birth at term. First trimester miscarriage was defined as pregnancy loss confirmed sonographically before 14^+0^ weeks’ gestational age using previously described criteria for cut‐off values.^23^, ^27^ Missed miscarriage was confirmed when there was an empty gestational sac with a mean sac diameter of >25 mm or more, if an embryo with a crown‐rump length (CRL) measurement of 7 mm or more was visualised without an embryonic heartbeat or if an embryonic heartbeat was absent irrespective of the size of the CRL where one had previously been observed.^23^, ^27^ A diagnosis of complete miscarriage was made when transvaginal ultrasound scan showed an empty uterus after a previous ultrasound scan had demonstrated an intrauterine pregnancy.^24^ A diagnosis of incomplete miscarriage was made when a transvaginal ultrasound demonstrated irregular heterogeneous tissue in the endometrial cavity in keeping with retained products of conception after a previous ultrasound scan had shown an intrauterine pregnancy.^24^ Second trimester miscarriage was defined as pregnancy loss under 24^+0^ weeks’ gestational age. Women with ongoing pregnancies were followed through to delivery. Any women experiencing antenatal delivery or neonatal complications (e.g. preterm birth, pre‐eclampsia, gestational diabetes) were excluded from the study. Women who experienced uncomplicated term deliveries were included as controls and were matched to cases for age, ethnicity and body mass index (BMI) at a ratio of approximately 1:1 miscarriages cases:control.

### DNA extraction and 16S rRNA gene amplicon sequencing

DNA extraction from vaginal swabs and sequencing was performed as previously described.^13^ Briefly, the V1‐V2 hypervariable regions of 16S rRNA genes were amplified for sequencing using a forward primer consisting of an Illumina i5 adapter (5′‐AATGATACGGCGACCACCGAGATCTACAC‐3′), an 8‐base pair (bp) bar code, a primer pad (forward, 5′‐TATGGTAATT‐3′) and the 28F primer (5′‐GAGTTTGATCNTGGCTCAG‐3′).^28^ The reverse fusion primer was constructed with an Illumina i7 adapter (5′‐CAAGCAGAAGACGGCATACGAGAT‐3′), an 8‐bp bar code, a primer pad (reverse, 5′‐AGTCAGTCAG‐3′) and the 388R primer (5′‐TGCTGCCTCCCGTAGGAGT‐3′). Sequencing was performed at RTL Genomics (Lubbock, TX, USA) using an Illumina MiSeq platform (Illumina Inc.). The MiSeq SOP Pipeline of the MOTHUR package was used to process the sequence data.^29^ Sequence alignment was performed using the Silva bacterial database (http://www.arb-silva.de/), and classification was performed using the RDP (Ribosomal Database Project) database reference sequence files and the Wang method.^30^ Determination of operational taxonomic unit taxonomies (phylum to genus) was performed using the RDP MultiClassifier script and USEARCH was used for species‐level taxonomies.^31^ To account for potential sequencing bias, data were resampled and normalised to the lowest read count.

### Statistical analysis

Statistical comparisons of continuous variables describing clinical and demographic parameters were calculated using analysis of variance (ANOVA) where data were normally distributed or Kruskal–Wallis test where data were not normally distributed. For categorical variables, Chi‐square test was applied.

Potential contaminants were identified as operational taxonomic units (OTUs) that were detected in at least half of negative control swabs at a proportional ratio of greater than 1:1 with the assumption that sequences found in negative controls are likely to have arisen from contamination from the sampling environment or extraction kit itself. These were removed and not considered for further downstream analyses.

Statistical comparisons between vaginal microbiota at genera and species level were performed within the Statistical Analysis of Metagenomic Profiles software package (STAMP).^32^ Hierarchical clustering analysis (HCA) using centroid linkage was performed using both bacterial genera and species level data in CLUSTVIS (https://biit.cs.ut.ee/clustvis) to classify samples into community groups on the basis of bacterial compositional similarity.

Chi‐square analyses were performed to test for significant differences in proportions of the vaginal community state type groups in the following groups: (2) All miscarriage samples versus viable control samples; (2) first trimester miscarriages versus viable pregnancies matched for gestational age and excluding longitudinal samples; (3) samples taken preceding diagnosis of first trimester miscarriage versus controls matched for gestational age and excluding longitudinal samples; (4) samples collected prior to diagnosis of second trimester miscarriage and controls; (5) samples taken from first trimester miscarriages versus viable pregnancies, excluding those with a reported bleeding score of greater than one, matched for gestational age and excluding longitudinal samples; (6) cases of missed miscarriages versus incomplete/complete miscarriage.

Differences in bacterial richness (species observed) and diversity (Inverse Simpson Index) between groups were analysed using one‐way ANOVA or the factorial Kruskal–Wallis test where appropriate. For pairwise comparisons in the same parameters in miscarriage and viable pregnancies, non‐parametric testing using Mann–Whitney *U* was applied.

To assess changes in the vaginal bacterial composition in the first trimester, longitudinal samples collected in a subset of women experiencing term delivery, first trimester and second trimester miscarriages were analysed. Vaginal bacterial community stability, or ‘transition index’ was calculated by dividing the number of different community group types observed by the number of samples collected from that individual throughout pregnancy.

## Results

**Figure 1:**
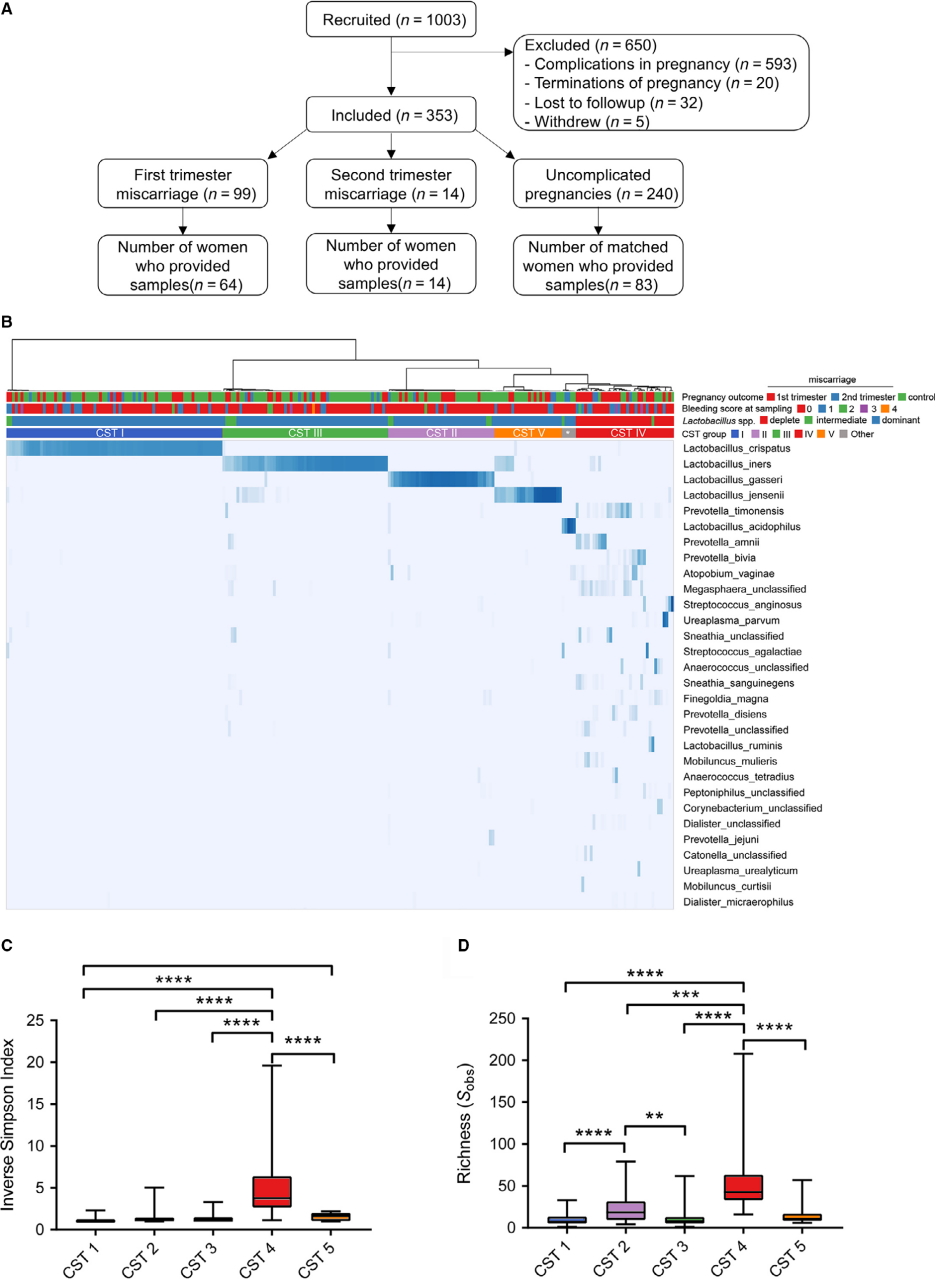
Study design and characterisation of vaginal bacterial composition in early pregnancy. (A) Flow chart describing study design and selection of cases and controls. (B) Hierarchal clustering of vaginal bacterial species data (top 30 species shown) in early pregnancy collected from women subsequently experiencing first trimester or second trimester miscarriages, or viable control pregnancies. Samples clustered into five major groups, four of which were dominated by *Lactobacillus* spp. (CST I, II, III, V) and one which was characterised by *Lactobacillus* spp. depletion (CST IV). *Indicates *Lactobacillus acidophilus* dominated. Both alpha diversity (C) and richness (D) were significantly higher in CST IV communities.

A total of 1003 women were recruited to the study. Those who underwent termination of pregnancy (*n *= 20), withdrew from the study (*n *= 5), were lost to follow up (*n *= 32) or subsequently experienced pregnancy complications other than miscarriage (e.g. preterm birth, pre‐eclampsia; *n *= 593) were excluded (Figure 1A). There were 99 first trimester miscarriages and 14 second trimester miscarriages. Of the 99 first trimester miscarriages, 64 had vaginal swabs taken at least once (sample *n *= 74). All women who had a second trimester miscarriage were sampled in the first trimester at least once (sample *n *= 24). Of the remaining 240 uncomplicated pregnancies, 83 were selected as control cases of term delivery matched for maternal age, ethnicity and BMI at a ratio of approximately 1:1 miscarriage cases:controls (sample *n *= 139, Table 1).

**Table 1:** Clinical and demographic characteristics of study cohort

	First trimester miscarriage *n* (%)	Second trimester miscarriage *n* (%)	Controls *n* (%)	*P*‐value*
**Number of women**	64	14	83	
**Number of samples**	74	24	139	
**Maternal age median, IQR (years)**	34.5 (28.0–38.8)	31.5 (24.0–35.0)	34.0 (29.0–38.0)	0.176
**BMI range, median, IQR (kg/m^2^)**	24.1 (22.6–27.0)	24.2 (21.1–27.4)	24.1 (21.7–26.6)	0.984
**Number of smokers, *n *(%)**	7 (10.9)	1 (7)	3 (3.6)	0.196
**Gravidity, *n *(%)**				
Primagravid	13 (20)	3 (21)	17 (20)	0.996
Multigravid	51 (80)	11 (79)	66 (80)	
**Parity, *n *(%)**				
Nulliparous	35 (55)	11 (79)	43 (52)	0.175
Multiparous	29 (45)	3 (21)	40 (48)	
**Previous miscarriage, *n* (%)**				
0	23 (36)	6 (43)	36 (43)	0.141
1–2	31 (48)	8 (57)	43 (52)	
≥3	10 (16)	0 (0)	4 (5)	
**Ethnicity (%)**				
White	48 (75)	6 (42)	61 (73)	0.140
Asian	8 (12.5)	5 (36)	11 (13)	
Black	8 (12.5)	3 (21)	11 (13)	
**Number of samples per gestational age group (%)**				
5–8 weeks	43 (58)	3 (13)	27 (19)	
8–10 weeks	26 (35)	8 (33)	57 (41)	
10–14 weeks	5 (7)	12 (50)	37 (27)	
>14 weeks	0	1 (4)	18 (13)	
**Number of samples with bleeding score> 1 at time of sampling**	10	0	0	
**Type of miscarriage**				
Missed	52 (81%)			
Incomplete/complete	12 (19%)			

BMI, body mass index; IQR, interquartile range; *N*, number.

* *P* values for maternal age calculated using ANOVA. For BMI, *P*‐values calculated using a Kruskal–Wallis test. For categorical variables, *P*‐values calculated using a Chi‐squared test.

A total of 237 swab samples were sequenced providing an average of 3267 reads per sample. After removing singletons and rare OTUs (<10 average reads per sample), 244 bacterial species were identified within the total sample cohort. Using hierarchical clustering of relative abundance data from the top 50 bacterial species (accounting for> 95% of all sequence reads), samples could be classified into five major groups similar to previously described vaginal community states types (CSTs) (Figure 1B).^12^ Note that for visualisation purposes, the top 30 taxa are presented in Figure 1B. Four groups were characterised by dominance of *Lactobacillus crispatus* (CST I), *Lactobacillus gasseri* (CST II), *Lactobacillus iners* (CST III) or *L. jensenii* (CST V), whereas CST IV was characterised by low relative abundance of *Lactobacillus* spp. and enrichment of facultative or strict anaerobic species often associated with BV. Consistent with this finding, the highest alpha diversity and richness was observed in CST IV type communities (Figure 1C,D). Additionally, two samples from two viable control patients and two samples from a patient experiencing second trimester miscarriage were dominated by *Lactobacillus acidophilus*, which were grouped as CST‐Other. Due to a lack of power, samples from these patients were excluded from additional comparative analyses, resulting in *n *= 81 for viable controls and *n *= 13 for second trimester miscarriage.

**Figure 2:**
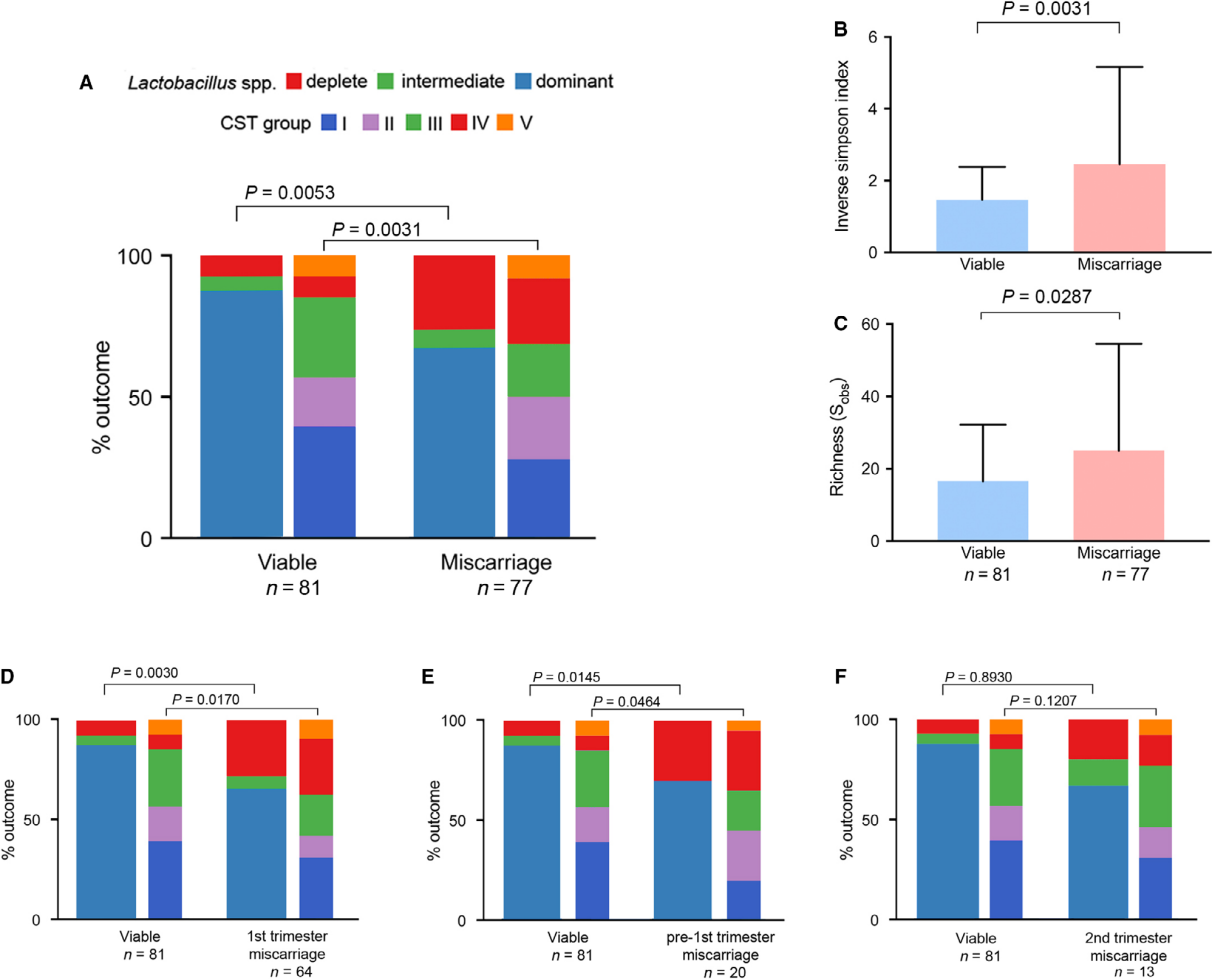
Vaginal *Lactobacillus* spp. depletion and high bacterial diversity is associated with first trimester miscarriage and precedes diagnosis. (A) Stacked bar chart showing that miscarriage (first and second trimester combined) is associated with a higher proportion of *Lactobacillus* spp.‐deplete, CST IV type vaginal microbiota community compositions as well as significantly greater vaginal (B) alpha diversity and (C) richness. (D) Sub‐analysis showed a similar relation in first trimester miscarriage which was also observed prior to diagnosis in the first trimester (E) but this did not reach statistical significance for second trimester miscarriage (F). CST, community state type.

**Figure 3:**
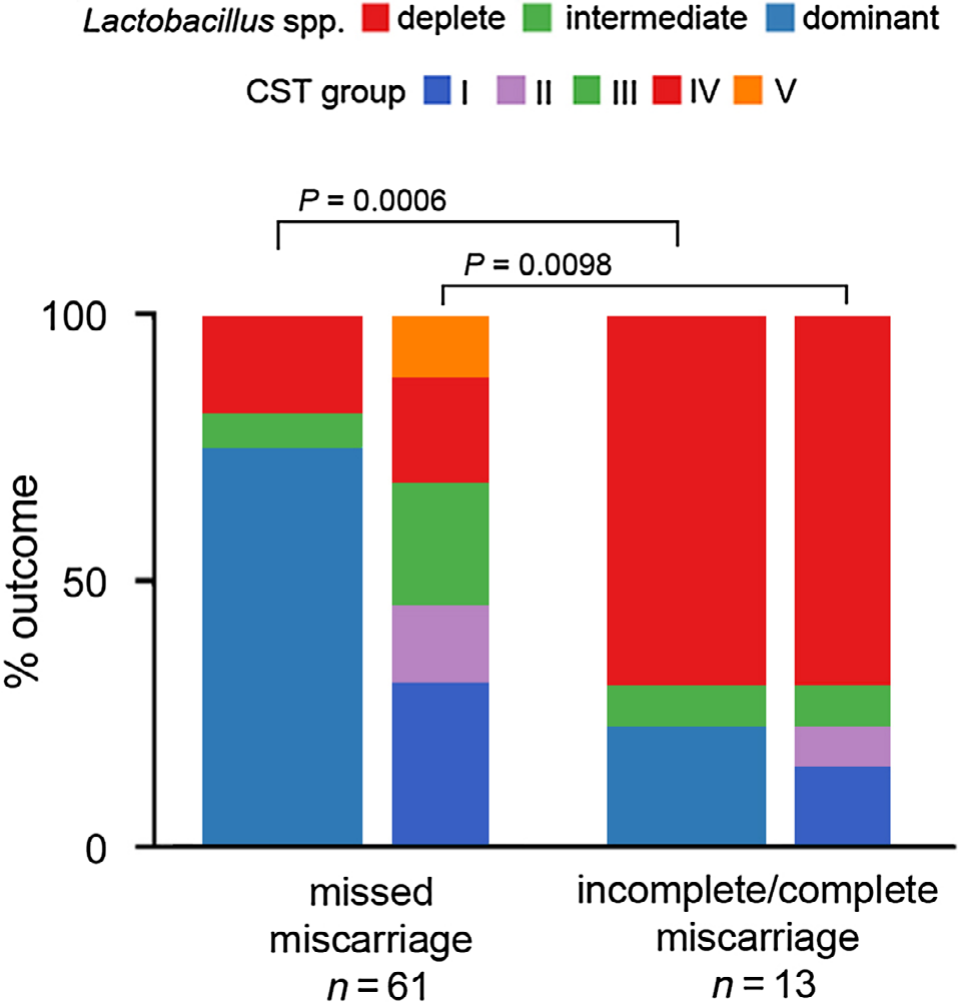
*Lactobacillus* spp.‐deplete vaginal microbial communities are more frequently associated with incomplete/complete miscarriage. Analysis of vaginal microbiota composition on the basis of miscarriage type shows that incomplete and/or complete miscarriages are associated with a significantly higher proportion of *Lactobacillus* spp.‐deplete, high diversity CST IV compared with missed miscarriages. CST, community state type.

Comparison of vaginal bacterial composition at genera level at the time of clinical confirmation of first or second trimester miscarriage (*n *= 77) with gestational‐aged matched samples from healthy controls (*n *= 81) showed that miscarriage is associated with *Lactobacillus* spp. depletion (*P *= 0.0053, Chi‐square) and at species level, a significantly higher proportion of samples dominated by CST IV (*P *= 0.031, Chi‐square) (Figure 2A). Consistent with this, bacterial alpha diversity, as estimated using the Inverse Simpson Index (*P *= 0.0031, Mann–Whitney), and richness (number of species observed, *P *= 0.0287, Mann–Whitney) were significantly higher in miscarriage samples than in matched control samples (Figure 2B,C). Vaginal bacterial composition from first trimester miscarriages (*n *= 64) was compared with viable control pregnancies (*n *= 81) matched for gestational age. A significant difference in the distributions of vaginal bacterial communities was observed between first trimester miscarriage samples and controls at both genera (*P *= 0.0030) and species level (*P *= 0.0170) (Figure 2D) with the former associated with *Lactobacillus* spp. depletion and an increased proportion of CST IV. This association was maintained when samples collected from participants reporting a vaginal bleeding score >1 were excluded (Figure S1). A similar relation was also observed in samples collected prior to miscarriage when the pregnancy appeared to be viable on ultrasound (*n *= 20) (Figure 3E) but not in samples taken prior to the second trimester (*n *= 13) (Figure 3F).

We next examined whether observed differences in vaginal bacterial composition were consistent across clinical miscarriage phenotypes, specifically missed miscarriages (*n *= 61) compared with incomplete and complete miscarriages (*n *= 13) (Figure 3). A significantly higher proportion of *Lactobacillus* spp. depleted (*P *= 0.0006) and CST IV (*P *= 0.0098) vaginal communities was observed in incomplete and complete miscarriages compared with missed miscarriages.

Comparison of vaginal bacterial community stability in longitudinal samples collected from control (*n *= 34) and miscarriage participants (first trimester miscarriage, *n *= 10: second trimester miscarriages, *n *= 9) showed no significant difference in the proportion of patients experiencing a transition from one genera (*P *= 0.8167) or CST (*P *= 0.5296) level composition group to another (Figure S2). This comparable stability was reflected by similar mean transition index scores between control and miscarriage participants (Figure S2).

## Discussion

### Main findings

First trimester is associated with reduced prevalence of *Lactobacillus* spp. dominated vaginal bacterial communities in the first trimester, irrespective of vaginal bleeding.

*Lactobacillus* spp.‐deplete, high diversity vaginal bacterial community composition precedes diagnosis of miscarriage.

Incomplete/complete miscarriage is associated with increased prevalence of *Lactobacillus* spp. depletion.

Early pregnancy vaginal bacterial stability in women who experience miscarriage is similar to those who have healthy viable pregnancies that deliver at term.

### Strengths and limitations

Strengths include the analysis of a large, well characterised cohort that includes longitudinal sampling prior to the miscarriage event and information regarding vaginal bleeding. In addition, the use of bacterial DNA sequencing approaches permits detection, identification and assessment of relative abundance of a broad range of commensal and pathogenic vaginal bacteria, including high resolution of key *Lactobacillus* spp. However, this does not capture information on other important microbiota constituents including viruses and fungi. Other limitations include the relatively low number of samples collected from women having second trimester miscarriage and those sampled prior to first trimester miscarriage diagnosis. Further, a lack of karyotyping information limits our ability to assess whether the observed association between first trimester miscarriage and *Lactobacillus* spp. depletion is similar between chromosomally normal and abnormal pregnancies. It is also important to note that our data are associative, and further studies are required to establish a causal relation between vaginal microbiota and miscarriage.

### Interpretation

Over half of all miscarriages are thought to be caused by structural or chromosomal abnormalities affecting the developing embryo.^1^ While the remaining causal factors have yet to be fully elucidated, an association between BV, as diagnosed using culture‐ and microscopy‐based methods, and increased risk of miscarriage have previously been reported.^8^, ^9^ Consistent with these findings, our study reports a positive relation between first trimester miscarriage and reduced vaginal *Lactobacillus* spp. abundance and increased prevalence of high diversity community compositions as assessed using 16S rRNA gene metataxonomics. Other recent studies using culture‐independent profiling methods have also identified an association between vaginal *Lactobacillus* spp. depletion and adverse reproductive health outcomes, including infertility, preterm birth and sexually transmitted diseases.^17^, ^18^, ^19^, ^20^, ^33^, ^34^ The protective role of *Lactobacillus* spp. in the lower reproductive tract is well described and can be partly attributed to lactic acid‐driven acidification of the vagina, which inhibits growth and colonisation of other pathogenic species.^15^ Furthermore, lactic acid can suppress production of inflammatory mediators in the vagina.^35^, ^36^, ^37^ Data from our cohort allow us to conclude that the protective role of *Lactobacillus* spp. extends into the early stages of pregnancy, where samples deplete of *Lactobacillus* spp. display increased richness and diversity and colonisation by potential pathogens including *Prevotella*, *Streptococcus*, *Peptoniphilus*, *Ureaplasma* and *Dialister* species. These bacteria have been shown to up‐regulate the expression of metalloproteinases^38^, ^39^ and pro‐inflammatory cytokines,^40^ while reducing the inhibitory effect of tissue inhibitors of metalloproteinases (TIMPs).^41^

Precise regulation of inflammatory and tissue remodelling in the upper reproductive tract is a critical aspect of early pregnancy events including implantation and placental trophoblast invasion.^42^, ^43^, ^44^ Vaginal microbiota may impact these pathways in a number of ways. Pathogen recognition and response by the maternal innate immune system following ascending vaginal infection accompanied by seeding and colonisation of the endometrium by pathogenic bacteria could drive untimely activation of inflammatory pathways that negatively modify endometrial receptivity and implantation. Consistent with this, ascending vaginal group B streptococcus (GBS; *Streptococcus agalactiae*) infection, which has recently been shown to be mediated by β‐catenin‐induced loss of vaginal epithelial barrier function and cellular detachment leading to exfoliation and subsequent bacterial ascension,^45^ is associated with increased rates of miscarriage, stillbirth and preterm birth.^46^ Further, it has recently been shown that, in mice, vaginal colonisation of bioluminescent *Escherichia coli* leads to ascending infection into the uterine cavity, activation of intrauterine inflammation and subsequent preterm birth and neonatal brain injury.^47^

There is also emerging evidence that despite previously being considered sterile,^48^ the uterus may harbour a functionally relevant microbiome.^49^, ^50^, ^51^, ^52^, ^53^, ^54^ The origin of the endometrial microbiome, and when it might be established, has yet to be determined, but seeding by vaginal bacteria is a plausible mechanism. Female subfertility is associated with bacterial vaginosis, which is often characterised by presence of an adherent vaginal polymicrobial biofilm.^8^ A structured polymicrobial *Gardnerella vaginalis* biofilm has been reported to be present in the endometrium of approximately half of patients presenting with bacterial vaginosis.^55^ It is also feasible that vaginal microbiota may modulate upper reproductive tract inflammatory pathways without direct colonisation of the endometrium. In mice, vaginal administration of bacterial lipopolysaccharide is sufficient to induce cervical remodelling, macrophage infiltration and subsequent preterm birth.^56^ Collectively, these data support a role for vaginal bacteria in an inflammatory aetiology that may underpin a proportion of miscarriage cases. This notion is supported by our findings showing that high diversity, CST IV communities, which we and others have previously shown to evoke local inflammatory activation,^18^, ^57^ are over‐represented in incomplete/complete miscarriages where the pregnancy is more likely to be expelled.

Due to the current study design, it is difficult to determine when *Lactobacillus* spp. depletion occurs in patients experiencing miscarriage, or what drives it mechanistically. Vaginal bleeding is a symptom of miscarriage,^24^ but it can also cause vaginal microbial instability and shifts towards *Lactobacillus* spp.‐deplete communities through alkalisation of the vaginal environment and provision of energy substrates that can be readily utilised by opportunistic colonisers.^13^ Concordant with this, we observed the highest proportions of *Lactobacillus* spp.‐deplete, high diversity community compositions, in incomplete/complete miscarriages where bleeding is a common symptom. However, the relation between *Lactobacillus* spp. depletion and first trimester miscarriage identified in our study appears to be independent of bleeding, as it remains following exclusion of samples with reported bleeding, and is seen where samples are taken prior to the miscarriage.

An alternative explanation for increased rates of vaginal *Lactobacillus* spp. depletion in miscarriage involves interaction between dysregulated hormonal signalling in miscarriage, which is associated with reduced estradiol and progesterone levels,^58^, ^59^ and microbial energy substrate availability. Estrogen production in pregnancy is thought to promote glycogen accumulation in vaginal epithelial cells, which can ultimately be used preferentially by *Lactobacillus* spp. as a primary carbon source.^12^ Therefore, low estrogen levels may fail sufficiently to promote *Lactobacillus* spp. dominance in some pregnancies that subsequently miscarry. Alternatively, vaginal microbiota communities associated with miscarriage may already be established prior to conception. Support for this second point is provided by our longitudinal analysis that indicates high levels of vaginal microbial stability in our patient cohort during early pregnancy. Further insight would be obtained through the analysis of pre‐conception samples.

It has been previously reported that bacterial vaginosis at 11–13 weeks’ gestation is associated with late miscarriage.^6^ In our study, a trend increase in the proportion of high diversity vaginal bacterial communities in the first trimester and subsequent second trimester miscarriage was observed, although this trend did not reach statistical significance. Second trimester miscarriages are associated with multiple aetiologies and therefore the small cohort included in this study may not have had sufficient power to detect an association with vaginal bacterial composition. Alternatively, this may be due to sampling predominately in the early first trimester, so that any later shifts towards *Lactobacillus* spp.‐deplete communities were not observed in this study.

## Conclusions

This study provides an insight into the first trimester vaginal microbiota composition and its relation with miscarriage. Through the application of 16S rRNA‐based metataxanomics, we show that *Lactobacillus* spp.‐deplete, high diversity vaginal bacterial communities are a risk factor for miscarriage. Unlike other causes of miscarriage, such as chromosomal abnormalities, the vaginal microbiome is a risk factor that can be potentially modifiable through the use of targeted antibiotic, prebiotic or probiotic treatments and other novel therapies, e.g. bacteriophage. Further studies designed to determine when and how high‐risk vaginal microbiota communities are established are needed to better understand this relation in order to elucidate further the role of infection in miscarriage and how treatment strategies might help us to prevent some miscarriages.

### Disclosure of interests

All authors declare that they have no competing interests. Completed disclosure of interest forms are available to view online as Supporting Information.

### Contribution to authorship

TB, PB and DAM conceived and designed the study. Patient recruitment and sample collection were undertaken by MA‐M, SB and HF. Experiments and data collection were performed by MA‐M, YSL and RM. Data analyses and interpretation were performed by MA‐M, AS, JRM, DT, TB, PRB and DAM. All figures and tables were generated by MA‐M and DAM. The manuscript was written by MA‐M and DAM and critically reviewed by all authors. All authors read and approved the final manuscript.

### Details of ethics approval

This study was approved by the National Health Service (NHS) National Research Ethics Service (NRES) Riverside Committee London (REC 14/LO/0199, approval date 15 January 2014).

### Funding

This study was supported by the Tommy’s National Centre for Miscarriage Research (grant P62774) and the UK National Institute for Health Research Biomedical Research Centre (grant P45272). TB, PRB and DAM are supported by the UK National Institute for Health Research Biomedical Research Centre (grant P45272), and the March of Dimes European Preterm Birth Research Centre at Imperial College London. SB was supported by NIHR CLAHRC NWL (Collaboration for Leadership in Applied Health Research & Care, North‐West London) and DAM was supported by the Medical Research Council (grant MR/L009226/1). The views expressed are those of the author(s) and not necessarily those of the NHS, the NIHR or the Department of Health. The Division of Integrative Systems Medicine and Digestive Disease at Imperial College London receives financial support from the National Institute of Health Research (NIHR) Imperial Biomedical Research Centre (BRC) based at Imperial College Healthcare NHS Trust and Imperial College London. This article is independent research funded by the NIHR BRC, and the views expressed in this publication are those of the authors and not necessarily those of the NHS, NIHR or the Department of Health.

### Acknowledgements

We would like to thank all the participants who took part in the study.

## Supporting information


**Figure S1** **.** Reduced *Lactobacillus* spp. is associated with miscarriage overall and first trimester miscarriage, irrespective of vaginal bleeding.
**Figure S2** **.** Longitudinal profiling of vaginal bacterial communities in early pregnancy.

## Data Availability

Public access to sequence data sets generated in this study along with accompanying metadata can be obtained from the Sequence Read Archive of the European Nucleotide Archive (PRJEB32479).
