# Supplementary material for: The association between vaginal bacterial composition and miscarriage: a nested case–control study
Source: BJOG. 2019 Oct 31;127(2):264–74. doi: 10.1111/1471-0528.15972 (PMC6972675; doi:10.1111/1471-0528.15972)
Supplement: Supplementary file 1 — Figure S1 . Reduced Lactobacillus spp. is associated with miscarriage overall and first trimester miscarriage, irrespective of vaginal bleeding. Figure S2 . Longitudinal profiling of vaginal bacterial communities in early pregnancy. [file BJO-127-264-s001.pdf]

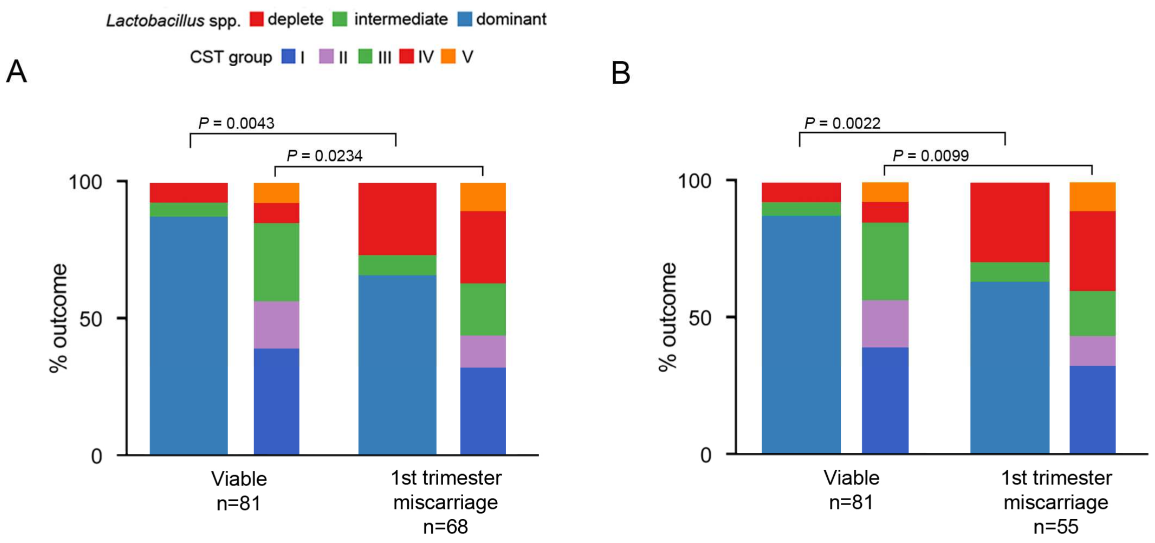

Figure S1. Reduced Lactobacillus spp. is associated with miscarriage overall and first trimester miscarriage, irrespective of vaginal bleeding.

(a) Reduced *Lactobacillus* spp. dominance of vaginal microbial communities was observed using genus (*P*=0·0043, chi-squared) and species level (P=0·0234, chi-squared) classifications in miscarriage samples after exclusion of samples collected with a bleeding score > 1. (b) Similar findings were found in first trimester miscarriages only at genus (P=0·0022, chi-squared) and species level (P=0·0099) groupings when similarly adjust for vaginal bleeding. CST = community state type.

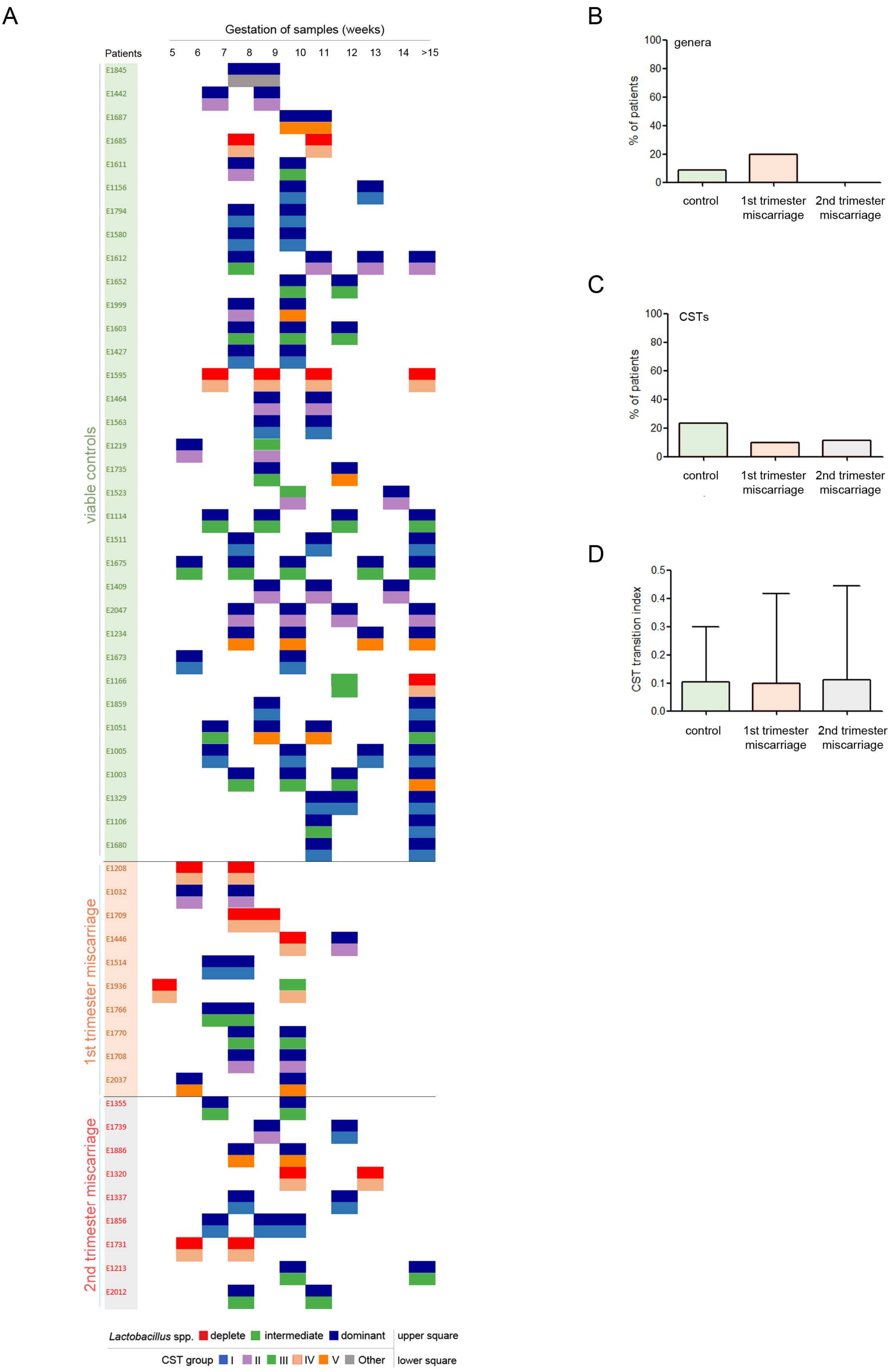
A
Gestation of samples (weeks)
Patients
5 6 7 8 9 10 11 12 13 14 >15
E1845
E1442
E1687
E1685
E1611
E1156
E1794
E1580
E1612
E1652
E1999
E1603
E1427
E1595
E1464
E1563
E1219
E1735
E1523
E1114
E1511
E1675
E1409
E2047
E1234
E1673
E1166
E1859
E1051
E1005
E1003
E1329
E1106
E1680
viable controls
E1208
E1032
E1709
E1446
E1514
E1936
E1766
E1770
E1708
E2037
1st trimester miscarriage
E1355
E1739
E1886
E1320
E1337
E1856
E1731
E1213
E2012
2nd trimester miscarriage
Lactobacillus spp. deplete intermediate dominant
upper square
CST group I II III IV V Other
lower square
B
genera
% of patients
100
80
60
40
20
0
control
1st trimester miscarriage
2nd trimester miscarriage
C
CSTs
% of patients
100
80
60
40
20
0
control
1st trimester miscarriage
2nd trimester miscarriage
D
CST transition index
0.5
0.4
0.3
0.2
0.1
0.0
control
1st trimester miscarriage
2nd trimester miscarriage

Figure S2. Longitudinal profiling of vaginal bacterial communities in early pregnancy.

(A) Profiling of vaginal microbial communities based on genera (upper square) and CST (lower square) level classifications for samples collected longitudinally from women subsequently experiencing term viable pregnancies (green, n=34), 1st trimester miscarriages (pink, n=10) and 2nd trimester miscarriages (grey, n=9), sampled on two or more occasions antenatally. Vaginal microbial community composition remained comparably stable throughout early pregnancy for all groups as assessed by examining the proportion of participants experiencing a transition from one (B) genera or (C) CST group to another. (D) This was reflected in similar mean transition index scores between CST groups. CST = community state type
